# Cross-talk between the transcription factor Sp1 and C/EBPβ modulates TGFβ1 production to negatively regulate the expression of chemokine RANTES

**DOI:** 10.1016/j.heliyon.2018.e00679

**Published:** 2018-07-04

**Authors:** Arisa Sakamoto, Rui Yamaguchi, Reona Yamaguchi, Shinji Narahara, Hiroyuki Sugiuchi, Yasuo Yamaguchi

**Affiliations:** a Graduate School of Medical Science, Kumamoto Health Science University, Kitaku Izumi-machi 325, Kumamoto 861-5598, Japan; b Department of Neuroscience, Graduate School of Medicine, Kyoto University, Yoshida-konoe-cho Sakyo-ku, Kyoto 606-8501, Japan

**Keywords:** Cell biology, Molecular biology

## Abstract

RANTES is a key chemokine for atherosclerosis, and obesity is associated with progression of atherosclerosis. Substance P (SP) increases glucose uptake and accumulation of lipids in adipocytes, and SP may upregulate RANTES expression. This study investigated the mechanism of RANTES expression by human M1 macrophages stimulated with SP.

SP upregulated RANTES protein expression, whereas aprepitant (an NK1R antagonist) blunted this response. Pretreatment of macrophages with BIRB796 (a combined p38γ/p38δ inhibitor) led to a significant decrease of RANTES expression. Next, we investigated the effect of several NK1R internalization factors on RANTES expression, including GRK2, β-arrestin 2, dynamin, ROCK, and TGFβ1. Exposure of macrophages to SP upregulated TGFβ1 expression. Silencing of β-arrestin 2 or GRK2 significantly enhanced the RANTES protein level after stimulation by SP, whereas TGFβ1/2/3 siRNA or dynasore (a dynamin inhibitor) decreased RANTES and Y-27632 (a ROCK inhibitor) had no effect. Surprisingly, silencing of transcription factor specificity protein 1 (Sp1) or inhibition of Sp1 activity by mithramycin led to significant upregulation of TGFβ1 protein and corresponding enhancement of RANTES expression (by ELISA or western blotting), whereas siRNA for C/EBPβ attenuated expression of both TGFβ1 and RANTES. Next, we investigated transcriptional cross-talk among Sp1 and C/EBPβ, TIF1β, or Fli-1 in relation to RANTES expression. Compared with TIF1β or Fli-1 siRNA, C/EBPβ siRNA showed significantly stronger inhibition of RANTES production by Sp1 siRNA-transfected macrophages after stimulation with SP.

In conclusion, transcription factor Sp1 engages in cross-talk with C/EBPβ and modulates TGFβ1 production to negatively regulate RANTES expression in macrophages stimulated with SP.

In conclusion, cross-talk between the transcription factor Sp1 and C/EBPβ modulates TGFβ1 production to negatively regulate expression of the atherogenic chemokine RANTES in SP-stimulated macrophages, while RANTES is upregulated by SP via the p38γδMAPK/C/EBPβ/TGFβ1 signaling pathway.

## 1 Introduction

Obesity is known to be an important risk factor for atherosclerosis [1]. Inflammatory cell recruitment to the intima is an initial step in atherosclerosis, and this process is triggered by chemokines produced by locally activated inflammatory cells. Accumulation of monocyte-derived macrophages in the vessel wall is a hallmark of the atherosclerotic process [2], and M1 macrophages are the dominant type linked to progression of atherosclerotic disease [3]. Regulated on activation, normal T cell expressed and secreted (RANTES) is produced by M1 macrophages [4] and has been shown to be involved in progression of atherosclerosis [5]. Insulin resistance associated with obesity is a major risk factor for cardiovascular disease. Substance P (SP) enhances insulin resistance at the adipocyte level [6] and increases glucose uptake, resulting in accumulation of lipids in adipocytes [7]. Thus, SP may promote expansion of the fat mass [8]. We previously reported that human visceral adipocytes obtained from subjects with a body mass index (BMI) >30 kg/m^2^ show higher production of SP than adipocytes from subjects with a BMI <30 kg/m^2^ [9], suggesting that visceral adipocytes could be a source of SP. Expression of various chemokines is upregulated by SP [10], and recruitment of inflammatory cells to the vascular intima by chemokines is essential to the development and progression of atherosclerosis. Therefore, SP may represent a link between visceral adipocytes and atherosclerosis. Accordingly, we investigated the mechanisms regulating expression of the atherogenic chemokine/ligand 5 CCL5/RANTES by human M1 macrophages after stimulation with SP.

Neurokinin 1 receptor (NK1R) is the receptor for SP. Recycling and resensitization of NK1R require its internalization in response to SP. Understanding the molecular mechanisms underlying SP-induced internalization of NK1R is important because of the essential role of signal transduction in trafficking of this receptor. During internalization, NK1R signaling not only occurs at the plasma membrane, but also the endosomal membrane. SP-induced internalization of NK1R is mediated by the GTPase dynamin [11, 12], which is essential for clathrin-mediated endocytosis of NK1R via membrane fission [13], and NK1R is colocalized with dynamin on the plasma membrane. The β-arrestins also serve as adaptors during SP-mediated internalization of NK1R [14].

Interestingly, transforming growth factor β1 (TGFβ1) has been shown to modulate phosphorylation of NK1R and delay its internalization after activation by SP [15, 16], with this delay of NK1R internalization leading to marked enhancement of SP-induced cellular signaling [17]. It also has been reported that SP upregulates TGFβ1 at the mRNA and protein levels [18], while TGFβ1 downregulates NK1 R gene expression [20]. Various transcription factors, such as specificity protein 1 (Sp1) and CCAAT-enhancer-binding protein β (C/EBPβ), induce TGFβ1 promoter activity [19, 20]. Therefore, we also investigated the influence of Sp1 and C/EBPβ on TGFβ1 expression by macrophages in response to SP stimulation.

## 2 Materials and methods

### 2.1 Ethics statement

Human peripheral blood samples were obtained from healthy volunteers and this study was approved by the Institutional Review Board of Kumamoto Health Science University. Written informed consent was obtained from all of the volunteers.

### 2.2 Chemicals and reagents

Human recombinant GM-CSF was obtained from Tocris Bioscience, Bristol, UK. Substance P (Peptide Institute Inc., Osaka, Japan), SB203580 (Wako, Kanagawa, Japan), aprepitant (Cayman Chemical, Ann Arbor, Michigan), PD98059 (Wako), BIRB796 (Axon Medchem, Groningen, Netherlands), U0216 (Promega Corporation, Madison, WI) and mithramycin (Abcam, Cambridge, UK) were employed to investigate the intracellular signaling pathways involved in RANTES production. The actions of all these reagents are summarized in Table 1.

**Table 1:** Functional characteristics of chemical agents used.

Chemical agents	Functions
Rottlerin	Protein kinase C inhibitor
TAPI-1	Disintegrin and metalloproteinase inhibitor
PDTC	The radical scavenger
Perifosine	Akt inhibitor
U0126	ERK1/2 inhibitor
Y-27632	ROCK inhibitor
Dynasore	Dynamin inhibitor
Aprepitant	Substance P/NK-1 receptor antagonists

Akt: Protein kinase B; ROCK: Rho-associated coiled-coil forming kinase; NK-1: Neurokinin 1; ERK: Extracellular signal-regulated kinase.

### 2.3 Isolation of adherent monocytes from peripheral blood mononuclear cells

Lymphocyte medium for thawing (BBLYMPH1) was obtained from Zen-Bio, Inc. (Research Triangle Park, NC). Peripheral blood mononuclear cells (PBMCs) were isolated as described previously [21]. Briefly, heparinized blood samples were obtained from nonsmoking healthy volunteers and were diluted 1:1 with pyrogen-free saline. Further, PBMCs were isolated immediately after collection using Lymphoprep gradients (Axis-Shield PoC As, Norway). Then, cells were suspended with BBLYMPH1 and incubated for 3 hrs. For monocyte isolation by plastic adherence, 1 × 10^6^ cells per well were distributed into 12-well plates (Corning Inc. Costar, NY, USA) and allowed to adhere in a 5% CO_2_ incubator at 37 °C for 2 hrs and washed 3 times with warm phosphate-buffered saline (PBS) to remove nonadherent cells. Then, monocytes were cultured in complete medium consisting of RPMI 1640 supplemented with 10% heat-inactivated fetal calf serum (FCS) and 10 × 10^3^ μg/L gentamicin at 37 °C in 5% CO_2_ humidified air. The adherent monocytes were recovered with a cell scraper. The purity of monocytes was evaluated by fluorescent staining with CD14-phycoerythrin (PE) mouse anti-human monoclonal antibody (Life Technologies, Staley Road Grand Island, NY) and fluorescence activated cell sorting (FACS) analysis. The recovery of monocytes was also evaluated by trypan blue staining and cells were counted using a Zeiss microscope (Jena, Germany). CD^14+^ monocytes had a purity of 85.43 ± 0.26% (mean ± SE, n = 60, 81.0–89.1). Consequently, CD^14+^ adherent macrophages expressing one of a broad range of plasma membrane receptors, such as mannose receptor (CD206), could be obtained from these cells [22]. Monocytes were resuspended in RPMI-1640 medium (Sigma-Aldrich, Oakville, Ontario, Canada) supplemented with 25 mM HEPES (Sigma-Aldrich), 100 mM/L L-glutamine (Sigma-Aldrich), 100 U/mL penicillin, and 100 μg/mL streptomycin (Sigma-Aldrich).

### 2.4 Induction of macrophages from adherent monocytes

CD14^+^ adherent monocytes were seeded at 1 × 10^6^ cells/mL into 12-well tissue culture plates containing RPMI-1640 medium with 10% FCS and 4 mM L-glutamine, and were incubated in the presence of 10 × 10^3^ ng/L recombinant human GM-CSF [23]. On day 1, 3 and 6, the cells were washed and then fresh media containing GM-CSF was added. GM-CSF-dependent macrophages (on 9 day of culture) were utilized as M1 macrophages in this study.

### 2.5 Enzyme-linked immunosorbent assay (ELISA) for RANTES

M1 macrophages were exposed to SP (0, 1 or 5 μM) with or without aprepitant (40 nM) for 6 hr and then protein levels of RANTES in whole-cell lysates were measured by ELISA (R&D Systems, Minneapolis, MN) with an anti-RANTES monoclonal antibody.

### 2.6 Identifying the intracellular signaling pathway for RANTES production

M1 macrophages were pretreated with PD98059 (10 μM), SB203580 (10 μM), BIRB796 (20 μM) or U0126 (60 μM) and then were stimulated for 6 hr with SP (5 μM). Then RANTES protein levels in whole-cell lysates were determined by ELISA (Abcam Inc.).

### 2.7 Extraction of RNA and amplification of RANTES

M1 macrophages (on day 9 of culture) were stimulated with SP (0, 5, 10 or 20 μM) for 6 hr, after which macrophages (1 × 10^6^ cells) were extracted with 1 mL of Isogen RNA kit (Nippon Gene, Toyama, Japan). Total RNA was isolated and precipitated according to the manufacturer's instructions, after which 200 ng of total RNA was reverse-transcribed using Oligo dT primer with a PrimeScript™ RT reagent Kit (Takara Bio, Shiga, Japan). Then the reverse-transcribed RNA was amplified by the polymerase chain reaction (PCR) using a TaKaRa PCR thermal cycler (Takara Bio, Shiga, Japan). Subsequently, human RANTES was amplified from genomic DNA by the reverse transcriptase-polymerase chain reaction (RT-PCR) using the following primers: 5′-CTACTCGGGAGGCTAAGGCAGGAA-3′ (forward for RANTES), 5′-GAGGGGTTGAGACGGCGGAAGC-3′ (reverse for RANTES), 5′-GTGGGGCGCCCCAGGCACCA-3′ (forward for β-actin), and 5′-CTCCTTAATGTCACGCACGATTTC-3′ (reverse for β-actin).

### 2.8 Luciferase reporter gene assay

The luciferase reporter gene assay was performed as described previously [24]. A RANTES promoter-luciferase reporter plasmid was obtained from Takara Bio Inc. (Shiga, Japan). Macrophages were transiently transfected with either this plasmid (RANTES) or the empty vector (Mock) using lipofectamine 2000 (Invitrogen, Carlsbad CA) on day 7 of culture [25]. Then macrophages were stimulated for 6 hr with substance P (0, 5, 10 or 20 μM) on day 9 of culture, and cellular extracts were prepared for the luciferase enzyme assay. To determine luciferase activity, 25 μL of Bright-Glo (Promega, Madison, WI) was added to the cells, and luminescence was measured using a TriLux luminescence counter (Perkin-Elmer Life Sciences, Rodgau, Germany) after incubation for 2 min at 37 °C.

### 2.9 Transfection of macrophages with small interfering RNA (siRNA)

Small interfering RNA for Sp1, β-arrestin 2, G-protein- coupled receptor kinase 2 (GRK2), transforming growth factor β1/2/3 (TGFβ1/2/3), C/EBPβ, transcriptional intermediary factor 1 β (TIF1β) or friend leukemia integration 1 (Fli-1) were obtained from Santa Cruz Biotechnology, Dallas, Texas. Transfection of M1 macrophages with siRNA for Sp1 (50 nM), β-arrestin 2 (50 nM), GRK2 (50 nM), TGFβ1/2/3 (50 nM), C/EBPβ (50 nM), TIF1β (50 nM) or Fli-1 (50 nM) was performed on days 7–8 of culture according to the manufacturer's protocol for Lipofectamine™ RNAiMAX (Life Technologies, Carlsbad, CA). Control siRNA-A (Santa Cruz Biotechnology) was utilized as the negative control for these experiments.

### 2.10 Effect of β-arrestin 2 siRNA, GRK2 siRNA, dynasore, Y-27632, or TGFβ1/2/3 siRNA on RANTES expression

To determine the role of β-arrestin 2, GRK2, dynamin, ROCK, and TGFβ1 in signaling during internalization of NK1R, we investigated the effect of β-arrestin 2 siRNA, GRK2 siRNA, TGFβ1/2/3 siRNA, or dynasore (a dynamin inhibitor) or Y-27632 (a ROCK inhibitor) on the expression of RANTES by macrophages after exposure to SP. M1 macrophages transfected with β-arrestin 2 siRNA (50 nM), GRK2 siRNA (50 nM), or TGFβ1/2/3 siRNA (50 nM) and macrophages pretreated with dynasore (10 μM) or Y-27632 (10 μM) were stimulated with SP (5 μM) for 6 hr, after which RANTES protein levels were measured by ELISA.

### 2.11 Effect of mithramycin (a gene-specific Sp1 inhibitor), Sp1 siRNA, C/EBPβ siRNA, or TGFβ1/2/3 siRNA on expression of TGFβ1 or RANTES

M1 macrophages (on day 9 of culture) were pretreated with mithramycin (1, 2, or 5 nM) for 6 hr or were transfected with Sp1 siRNA, C/EBPβ siRNA, or TGFβ1/2/3 siRNA. Then the cells were stimulated with SP (5 μM) for 6 hr, after which latency-associated peptide (LAP: TGFβ1) or RANTES proteins were detected by ELISA.

### 2.12 Effect of silencing Sp1, C/EBPβ, TIF1β, or Fli-1 on RANTES expression by M1 macrophages after stimulation with SP

M1 macrophages were transfected with siRNA for Sp1, C/EBPβ, TIF1β, or Fli-1 and then were stimulated with SP (5 μM) for 6 hr, after which RANTES protein was determined in whole-cell lysates by ELISA.

### 2.13 Western blotting for TGFβ1

M1 macrophages were transfected with siRNA for Sp1, TGFβ1/2/3 or C/EBPβ. Then TGFβ1 protein production by the transfected cells was detected by western blotting of whole-cell lysates with an anti-mouse monoclonal antibody for TGFβ1 (Santa Cruz Biotechnology, Santa Cruz, CA). Whole-cell lysates of M1 macrophages stimulated with human recombinant TNFα (1 ng) for 6 hr were utilized as the positive control. Glyceraldehyde 3-phosphate dehydrogenase (GAPDH) was also detected by western blotting with an anti-GAPDH antibody (Santa Cruz Biotechnology).

### 2.14 Statistical analysis

Results are expressed as the mean ± SE. Analysis of variance and the *t*-test for independent means were used to assess differences between multiple groups and differences between two groups, respectively. When the F ratio was found to be significant, mean values were compared by using a post hoc Bonferroni test. A probability (P) value < 0.05 was considered to indicate significance in all analyses.

## 3 Results

**Fig. 1:**
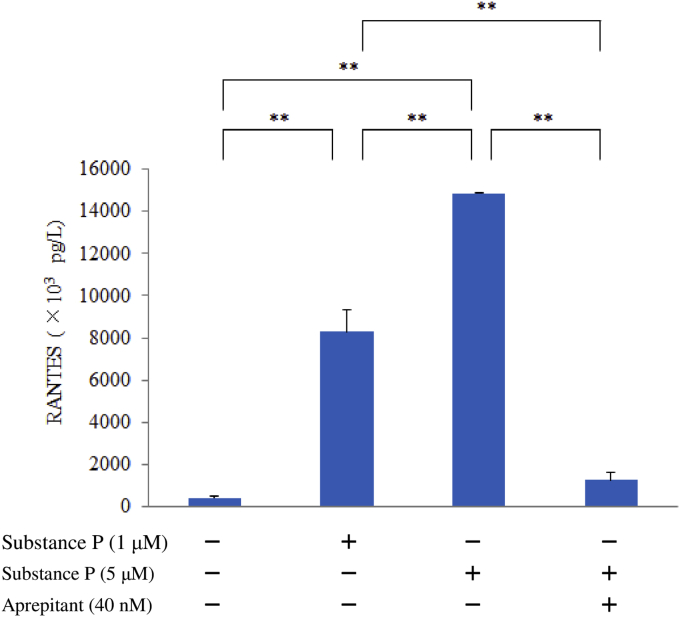
Effect of SP on RANTES production by M1 macrophages. M1 macrophages (day 9 of culture) were stimulated with SP (5 μM) for 6 hr in the presence or absence of aprepitant. Then RANTES protein levels in whole-cell lysates were determined by ELISA. Data were obtained using macrophages from three individuals in each group and represent the mean ± SE. **P < .01 (with Bonferroni's correction).

**Fig. 2:**
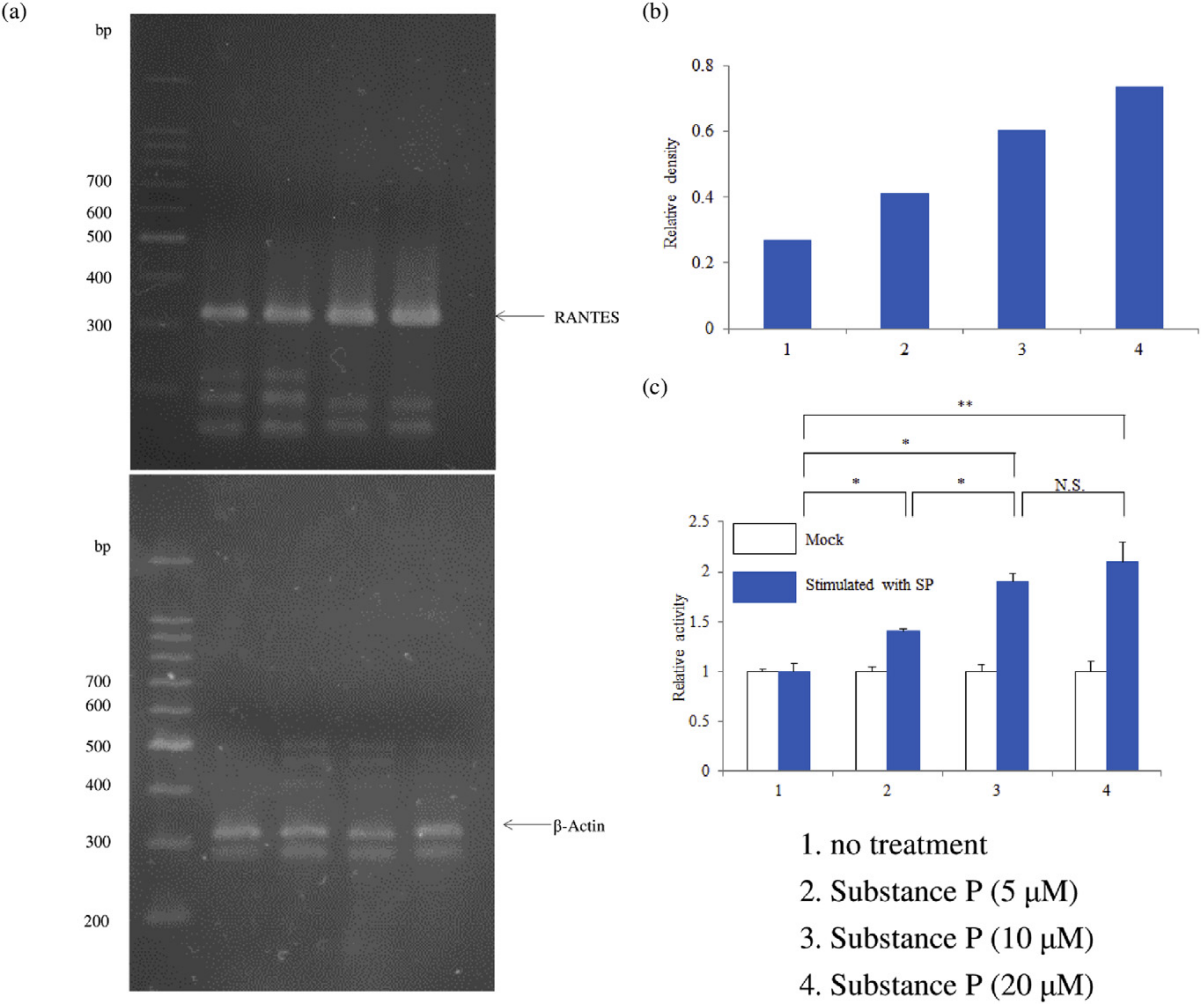
Expression of RANTES mRNA and luciferase enzyme assay. Expression of RANTES mRNA after stimulation of macrophages with SP was detected by the reverse transcription polymerase chain reaction (RT-PCR). The density of the RANTES band was normalized to that of the β-actin band. (a) Representative RT-PCR (b) Densitometric data. Luciferase activity in unstimulated cells transfected with the reporter plasmid alone (Mock) was set as 1.0. (c) Relative luciferase activity. Data were obtained using macrophages from three individuals in each group and represent the mean ± SE. **P < .01; *P < .05 (with Bonferroni's correction); N.S., not significant.

**Fig. 3:**
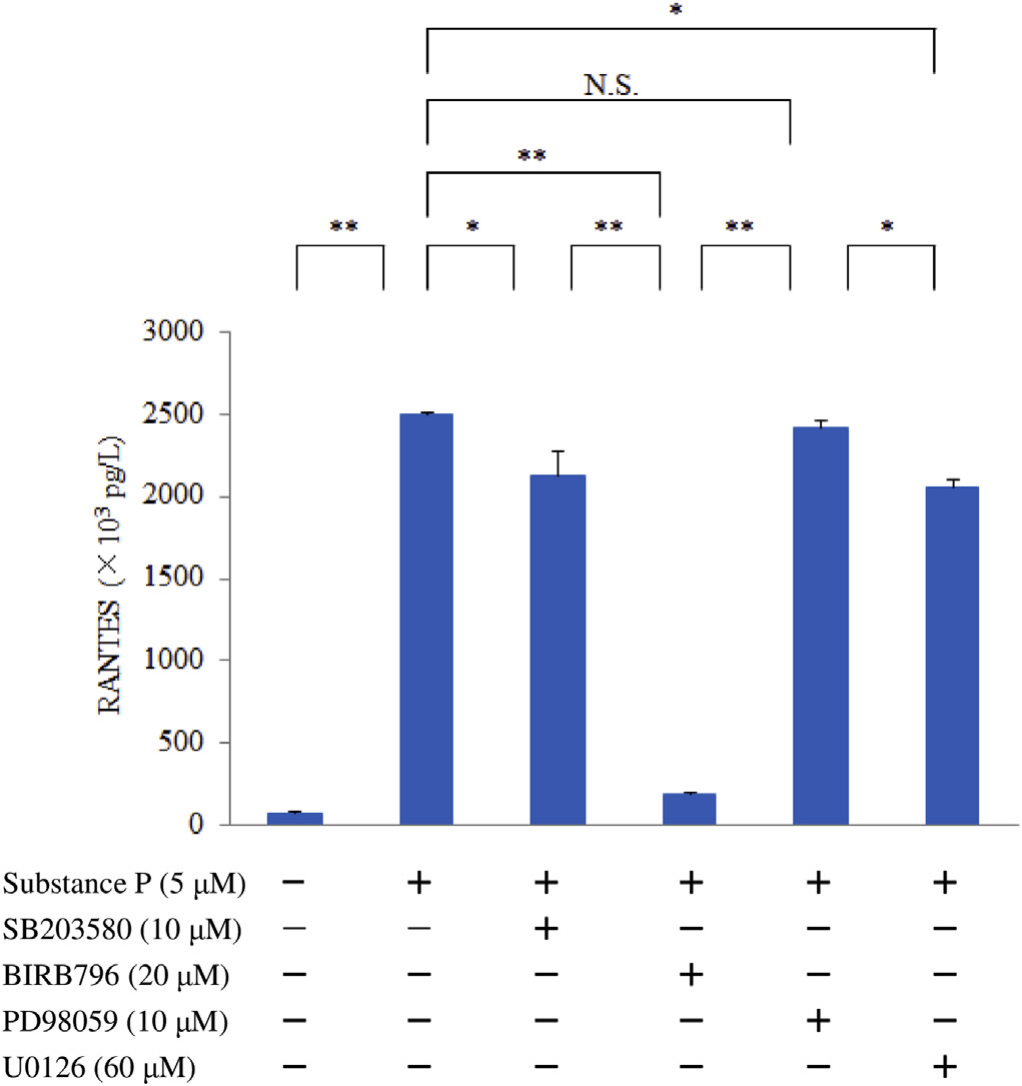
Effect of PD98059, SB203580, BIRB796, or U0126 on RANTES production by M1 macrophages stimulated with SP. M1 macrophages day 9 of culture were pretreated with PD98059, SB203580, BIRB796, or U0126 and then were stimulated with SP for 6 hr, after which RANTES protein levels in whole-cell lysates were determined by ELISA. Data were obtained by using cells from three different donors in each group and represent the mean ± SE. **P < .01; *P < .05 (with Bonferroni's correction); N.S., not significant.

**Fig. 4:**
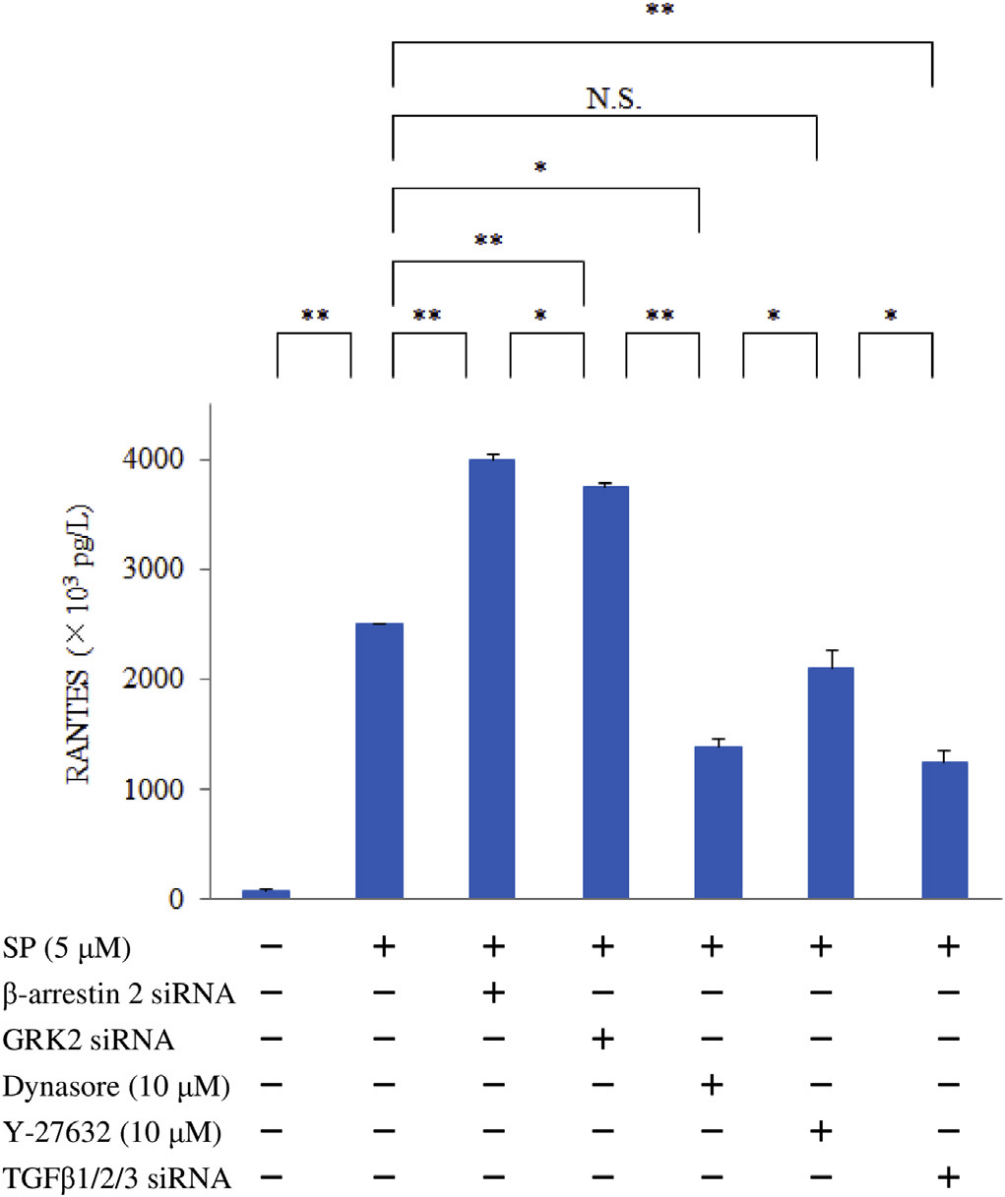
Effect of β-arrestin 2 siRNA, GRK2 siRNA, dynasore, Y-27632, or TGFβ1/2/3 siRNA on RANTES expression by M1 macrophages after exposure to SP. M1 macrophages (day 9 of culture) transfected with β-arrestin 2 sRNA, GRK2 siRNA, or TGFβ1/2/3 siRNA and macrophages pretreated with dynasore (10 μM) or Y-27632 (10 μM) were stimulated with SP (5 μM) for 6 hr, after which RANTES protein levels were measured by ELISA. Data were obtained by using cells from three different donors in each group and represent the mean ± SE. **P < .01; *P < .05 (with Bonferroni's correction).

Stimulation with SP upregulated RANTES production by human M1 macrophages in a concentration-dependent manner compared with untreated control cells. This response was blunted by aprepitant, which is an NK1R antagonist (Fig. 1). SP caused upregulation of the relative level of RANTES mRNA. In the luciferase reporter gene assay, relative luciferase activity was increased in a concentration-dependent manner by stimulation with SP (Fig. 2). Interestingly, pretreatment with BIRB796 (a p38γ/p38δ MAPK inhibitor) significantly decreased the expression of RANTES protein by SP-stimulated macrophages, while neither SB203580 (a p38α/p38β inhibitor) nor PD98059 (an ERK1 inhibitor) affected RANTES expression after SP stimulation. High concentrations of U0126 (an ERK1/2 inhibitor) also had no effect on RANTES production (Fig. 3). Next, we investigated the role of β-arrestin 2, GRK2, dynamin, ROCK, and TGFβ1, which are associated with NK1R internalization and trafficking, in signal transduction associated with RANTES expression. Accordingly, we assessed the effect of β-arrestin 2 siRNA, GRK2 siRNA, or TGFβ1/2/3 siRNA, dynasore (a dynamin inhibitor) or Y-27632 (a ROCK inhibitor) on RANTES protein levels after exposure of macrophages to SP. Unexpectedly, β-arrestin 2 siRNA and GRK2 siRNA significantly upregulated RANTES protein production, whereas TGFβ1/2/3 siRNA or dynasore attenuated it and Y-27632 had no effect (Fig. 4).

**Fig. 5:**
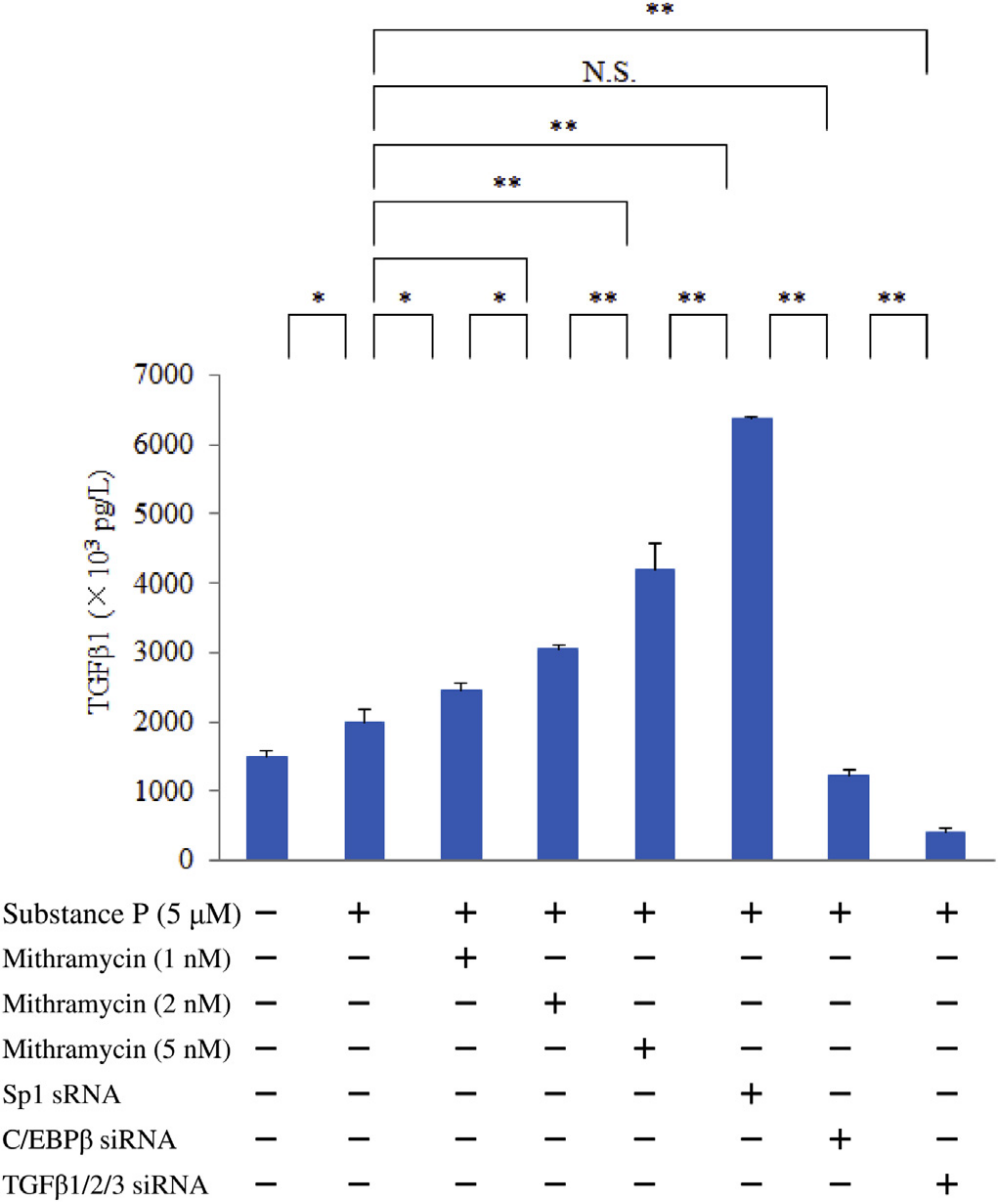
Effect of mithramycin, Sp1 siRNA, C/EBPβ siRNA, or TGFβ1/2/3 siRNA on TGFβ1 expression by SP-stimulated M1 macrophages. M1 macrophages (day 9 of culture) pretreated with mithramycin (0, 1, 2, or 5 nM) and macrophages transfected with Sp1 siRNA, C/EBPβ siRNA, or TGFβ1/2/3 siRNA were stimulated with SP (5 μM) for 6 hr. Then TGFβ1 protein levels in whole-cell lysates were measured by ELISA. Data were obtained by using cells from three different donors in each group and represent the mean ± SE. **P < .01; *P < .05 (with Bonferroni's correction).

**Fig. 6:**
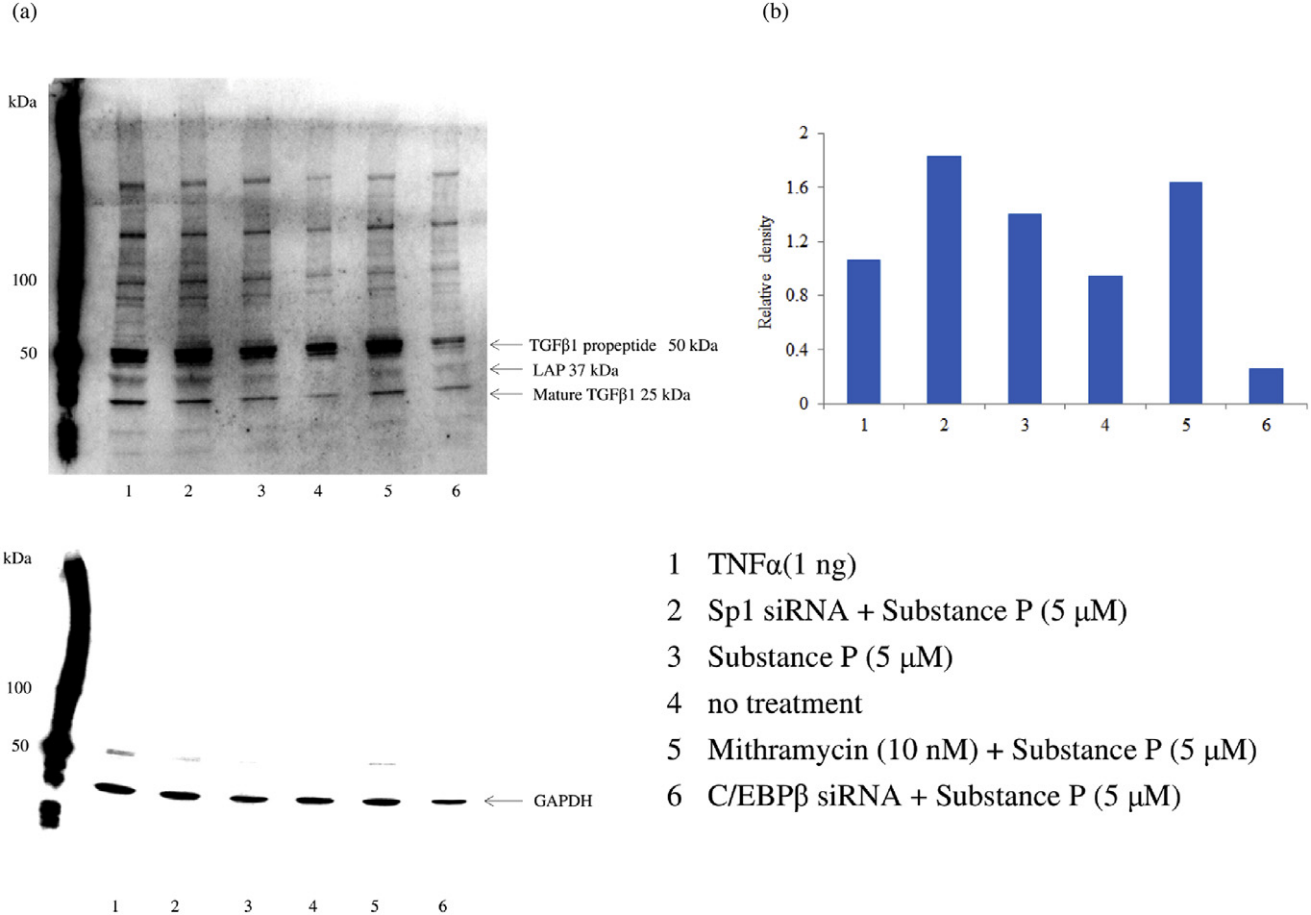
Western blotting for detection of TGFβ1. After pretreatment of M1 macrophages (day 9 of culture) with mithramycin (5 nM) or transfection with Sp1 siRNA, C/EBPβ siRNA or TGFβ1/2/3 siRNA, the cells were stimulated with SP (5 μM) for 6 hr. Then TGFβ1 protein was detected by western blotting of whole-cell lysates. Whole-cell lysates of M1 macrophages stimulated with recombinant TNFα (1 ng) for 6 hr were utilized as the positive control. The density of the TNFβ1 band was normalized to that of GAPDH. Samples were tested in triplicate and three separate experiments were performed. (a) Representative western blot. (b) Densitometry data.

Interestingly, silencing of Sp1 significantly enhanced the TGFβ1 protein level after exposure of macrophages to SP. We also investigated the effect of mithramycin (an inhibitor of DNA binding by Sp1 family members) on TGFβ1 expression after SP stimulation, revealing that inhibition of Sp1 activity by mithramycin significantly increased the TGFβ1 protein level in a concentration-dependent manner. Surprisingly, C/EBPβ siRNA attenuated TGFβ1 production, unlike Sp1 siRNA (Fig. 5). Western blotting also demonstrated the enhancement of TGFβ1 expression by Sp1 siRNA or mithramycin, but not by C/EBPβ siRNA (Fig. 6).

**Fig. 7:**
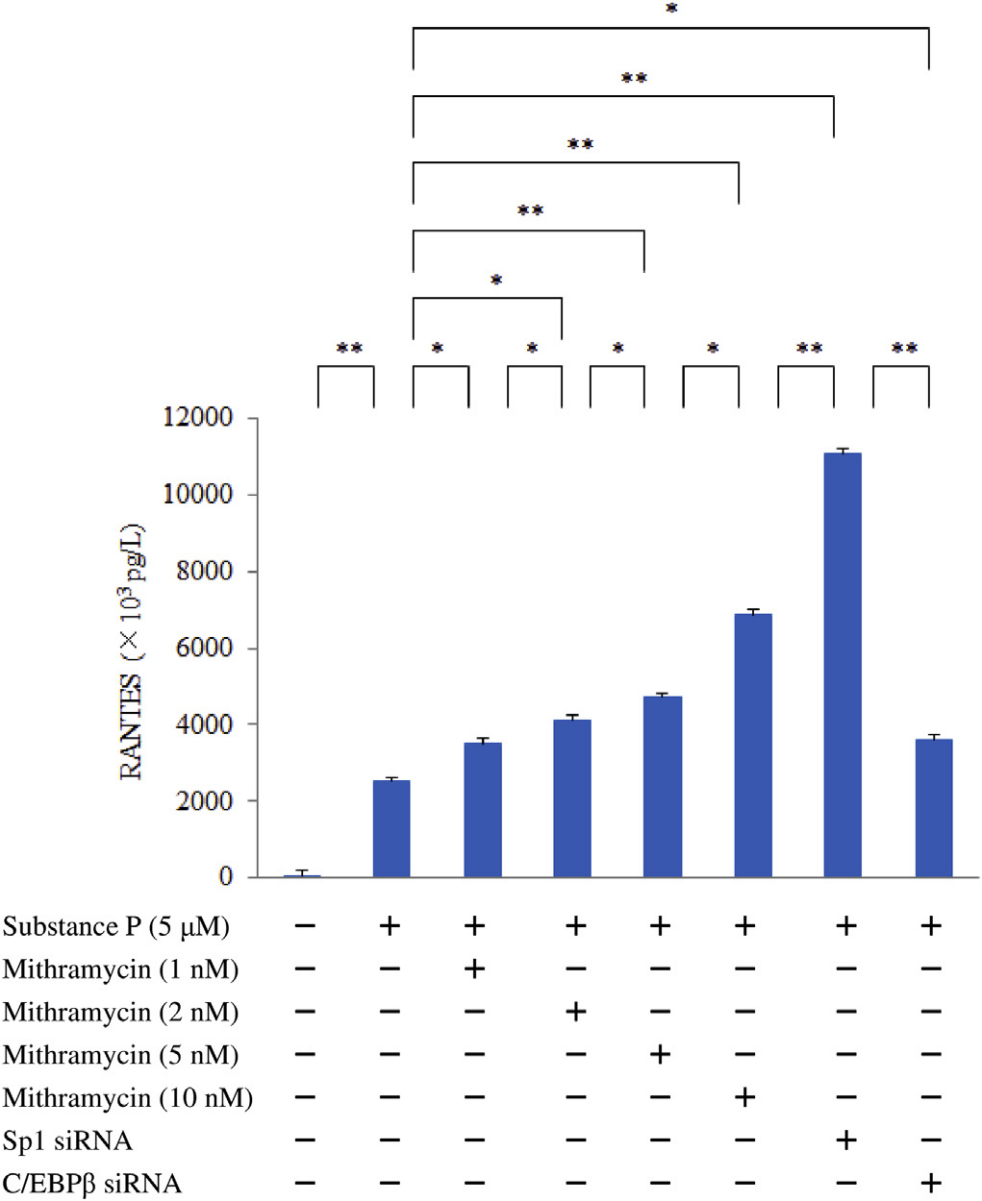
Effect of mithramycin, Sp1 siRNA, C/EBPβ siRNA, or TGFβ1/2/3 siRNA on RANTES expression by M1 macrophages exposed to SP. M1 macrophages (day 9 of culture) pretreated with mithramycin (0, 1, 2, or 5 nM) and macrophages transfected with Sp1 siRNA, C/EBPβ siRNA, or TGFβ1/2/3 siRNA were stimulated with SP (5 μM) for 6 hr. Then RANTES protein levels in whole-cell lysates were measured by ELISA. Data were obtained by using cells from three different donors in each group and represent the mean ± SE. **P < .01; *P < .05 (with Bonferroni's correction).

**Fig. 8:**
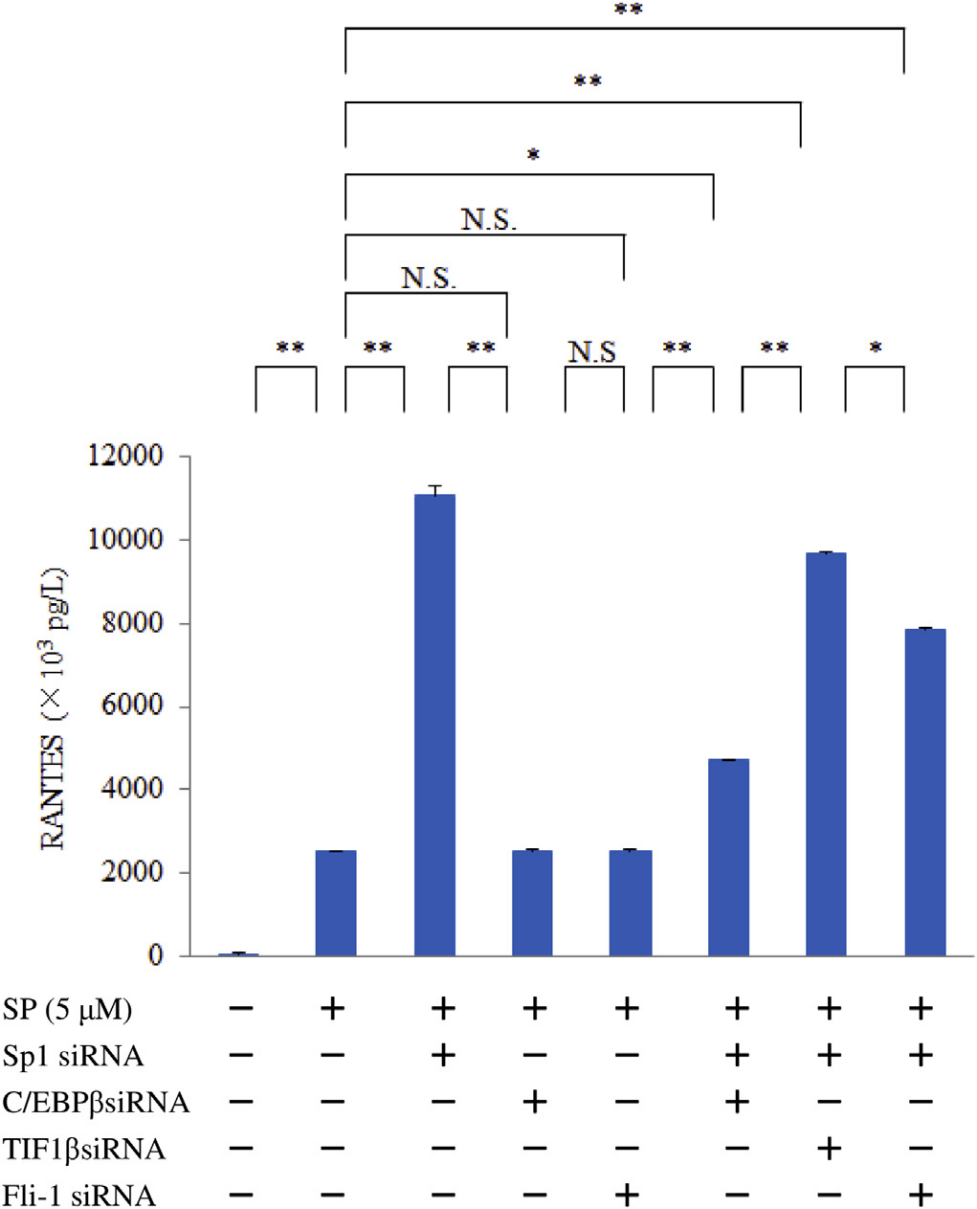
Effect of silencing Sp1, C/EBPβ, TIF1β, or Fli-1 on RANTES expression by SP-stimulated M1 macrophages. M1 macrophages (day 9 of culture) transfected with Sp1 siRNA, C/EBPβ siRNA, TIF1β siRNA, or Fli-1 siRNA or macrophages transfected with Sp1 siRNA+ C/EBPβ siRNA, Sp1 siRNA+ TIF1β siRNA, or Sp1 siRNA+ Fli-1 siRNA were stimulated with SP for 6 hr. Then RANTES protein levels were measured by ELISA. Data were obtained by using cells from three different donors in each group and represent the mean ± SE. **P < .01; *P < .05 (with Bonferroni's correction); N.S. not significant.

**Fig. 9:**
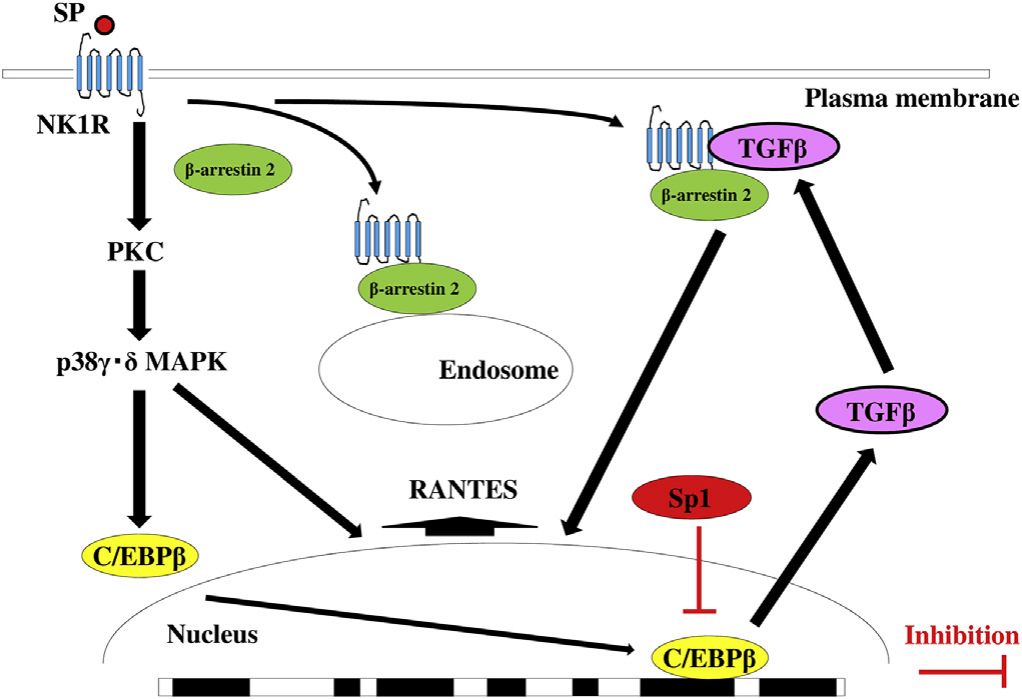
Proposed mechanisms by which transcription factor specificity protein 1 modulates TGFβ1 production to negatively regulate RANTES expression in macrophages stimulated with substance P. Substance P (SP) upregulates RANTES expression via the p38γδMAPK signaling pathway. TGFβ1 delays SP-induced internalization of NK1R and thus enhances signaling. SP also upregulates TGFβ1 by activation of the transcription factor C/EBPβ. In contrast, specificity protein 1 (Sp1) suppresses induction of TGFβ1 via cross-talk with C/EBPβ, thus influencing RANTES expression by macrophages after stimulation with SP.

Sp1 siRNA significantly upregulated RANTES protein production by macrophages after exposure to SP compared to untreated control cells. Mithramycin also enhanced the RANTES protein level in a concentration-dependent manner, but silencing of C/EBPβ attenuated RANTES production in response to SP (Fig. 7). We also investigated the role of cross-talk among transcription factors (between Sp1 and C/EBPβ, TIF1β, or Fli-1) in RANTES expression by performing double transfection. Compared with TIF1β siRNA or Fli-1 siRNA, we found that C/EBPβ siRNA significantly inhibited RANTES production by Sp1 siRNA-transfected macrophages after SP stimulation (Fig. 8). Fig. 9 displays the proposed protein-protein interactions and signaling pathways addressed in this study.

## 4 Discussion

The role of SP in regulating expression of the atherogenic chemokine RANTES by human M1 macrophages is poorly understood. The present study demonstrated that SP upregulated RANTES production by macrophages in a concentration-dependent manner, whereas aprepitant (which inhibits NK1R) blunted this response. SP has previously been shown to increase phosphorylation of p38MAPK and ERK1/2 [26]. However, we found that RANTES production in response to SP stimulation was significantly blunted by BIRB796 (a p38γ/p38δ inhibitor).

Delayed internalization of NK1R has been shown to enhance its signaling [17]. TGFβ1 has been reported to delay SP-induced NK1R internalization [16], resulting in enhancement of signaling by SP [17]. This study also showed that SP upregulated TGFβ1 in macrophages exposed to SP with corresponding enhancement of RANTES protein expression. Interestingly, this enhancement of RANTES production was blunted by TGFβ1/2/3 siRNA, which implies that TGFβ1 promotes RANTES expression via SP-mediated activation of NK1R. Both transcription factor Sp1 and C/EBPβ are known to be promoters of TGFβ1 [19, 20]. Therefore, we investigated the effect of Sp1 or C/EBPβ on TGFβ1 protein production by macrophages stimulated with SP. Unexpectedly, silencing of Sp1 significantly upregulated TGFβ1 expression and consequently increased the RANTES protein level in SP-stimulated macrophages. Moreover, inhibition of Sp1 activity by mithramycin augmented both TGFβ1 and RANTES protein expression in a concentration-dependent manner. Mithramycin is a gene-selective Sp1 inhibitor that binds to GC-rich DNA sequences and displaces Sp1 [27] or modulates Sp1 protein by proteasome-dependent degradation [28]. Several cytokine/chemokine genes are induced or repressed by the transcription factor C/EBPβ. In the present study, we found that TGFβ1 production was inhibited by C/EBPβ siRNA. Activated p38 MAPK regulates C/EBPβ via phosphorylation [29], and this study revealed that SP promoted TGFβ1 expression via the NK1R/p38γδMAPK/C/EBPβ signaling pathway. Thus, Sp1 and C/EBPβ had opposing effects on TGFβ1 expression. Cross-talk among transcription factors pathways is extremely complex, with tandem combinations of transcription factors variously being inert or having additive, synergistic, or antagonistic effects. C/EBP-β binds to a number of response elements and forms heteromeric complexes with other transcription factors such as Sp1. Indeed, the C/EBPβ promoter contains a TATA box and binding sites for several transcription factors that regulate C/EBPβ mRNA expression, including C/EBPβ itself [30], signal transducer and activator of transcription 3 (STAT3) [31], and Sp1 [32]. In this study, we demonstrated that C/EBPβ siRNA attenuated TGFβ1 protein production, whereas it was increased by Sp1 siRNA. Moreover, it has been reported that C/EBPβ shows synergistic transcriptional activation of the CYP2D5 P-450 gene with Sp1 [33]. On the other hand, cross-talk between Sp1 and C/EBPβ has the opposite effect on induction of tumor suppressor ARF [34].

The human RANTES promoter region contains a wide variety of transcription factor-binding sites, including sites for CCAAT-enhancer-binding protein (C/EBP) and friend leukemia integration 1 (Fli-1) [35]. Transcriptional intermediary factor 1 beta (TIF1β) was reported to act as a coactivator of C/EBPβ [36]. Therefore, we investigated the influence of transcriptional cross-talk between Sp1 and C/EBPβ, Fli-1, or TIF1β on RANTES expression by SP-stimulated macrophages. Double transfection of macrophages with Sp1 and C/EBPβ siRNA showed a significantly greater inhibitory effect on RANTES production after exposure to SP, compared with transfection of Fli-1 or TIF1β siRNA. Importantly, double transfection of macrophages with Sp1 and C/EBPβ siRNA reduced the TGFβ1 protein level after exposure to SP. These results suggest that SP1 and C/EBPβ are negative and positive regulators of TGFβ1 expression, respectively, implying antagonistic cross-talk between these factors.

Obesity is associated with vascular diseases that are often attributed to oxidative stress [37], and prolonged endoplasmic reticulum stress leads to increased production of reactive oxygen species. C/EBPβ is highly expressed by macrophages and regulates NADPH oxidases, which are involved in one of the basic pathogenetic processes of atherosclerosis. The present study revealed that SP promoted TGFβ1 expression via the NK1R/p38γδ MAPK/C/EBPβ signaling pathway, resulting in enhanced production of RANTES through TGFβ1-induced delay of NK1R internalization and consequent enhancement of SP signaling. Interestingly, we found that Sp1/C/EBPβ cross-talk inhibited the increase of RANTES expression in response to SP, which may imply a link between obesity and regulation of atherogenic RANTES expression via C/EBPβ.

In conclusion, the transcription factor Sp1 is involved in cross-talk with C/EBPβ and modulates TGFβ1 production to negatively regulate RANTES expression by SP-stimulated macrophages.

## Declarations

### Author contribution statement

Arisa Sakamoto: Conceived and designed the experiments; Performed the experiments; Wrote the paper.

Rui Yamaguchi: Performed the experiments; Contributed reagents, materials, analysis tools or data.

Reona Yamaguchi: Conceived and designed the experiments; Analyzed and interpreted the data.

Shinji Narahara, Hiroyuki Sugiuchi: Performed the experiments.

### Funding statement

This work was partly supported by a Kumamoto Health Science University special fellowship grant (No. 27-A-1 and 2016-C-15).

### Competing interest statement

The authors declare no conflict of interest.

### Additional information

No additional information is available for this paper.
